# A Rare Case of Pembrolizumab-Associated Graves’ Disease

**DOI:** 10.7759/cureus.34696

**Published:** 2023-02-06

**Authors:** Sura Alqaisi, Ali Rahman

**Affiliations:** 1 Internal Medicine, Memorial Healthcare, Pembroke Pines, USA; 2 Internal Medicine, Northwell Health at Mather Hospital, Port Jefferson, USA

**Keywords:** immune-related adverse events, immune checkpoint inhibitors, hyperthyroidism, thyrotoxicosis, graves’ disease, keytruda®, pembrolizumab

## Abstract

Immune checkpoint inhibitors (ICPis), such as pembrolizumab (Keytruda®), are associated with the development of several immune-related adverse events (irAEs). Thyroid dysfunction is a common endocrine irAE associated with pembrolizumab; however, Graves’ disease induced by pembrolizumab is extremely rare. Few cases of this condition have been reported in the literature. Here, we report the case of a 50-year-old patient who presented with thyrotoxicosis that was attributed to Graves’ disease secondary to pembrolizumab therapy.

## Introduction

Tumor cells can evade tumor immune surveillance via a variety of mechanisms. One such immune subversion mechanism is the expression of immune checkpoint molecules, including cytotoxic T lymphocyte-associated protein 4 (CTLA-4) and programmed cell death protein 1 (PD-1), on T lymphocytes. The expression of these molecules limits T-cell activation and, thus, suppresses the antitumor immune responses [1,2].

Immune checkpoint inhibitors (ICPis), including anti-PD-1, anti-programmed death-ligand 1 (PD-L1), and anti-CTLA-4 monoclonal antibodies, upregulate the antitumor immune responses by blocking the PD-1 or CTLA-4 signaling pathways. The development of these immunotherapeutic agents has significantly improved the management and prognosis of several malignancies. However, immune checkpoint inhibitors, such as pembrolizumab (Keytruda®), have been associated with the development of immune-related adverse events (irAEs) in some cancer patients [2,3].

Graves’ disease as an irAE secondary to pembrolizumab therapy has been rarely reported in the literature. This case report presents a patient who developed Graves’ disease after initiating pembrolizumab therapy for colon cancer.

## Case presentation

A 50-year-old male presented to the emergency department of our hospital with complaints of fever, chills, nausea, and vomiting for the past week. He also complained of occasional abdominal pain and unintentional weight loss of 10 lbs over one month. The patient had a history of colon cancer with lymph node metastasis, for which he had undergone tumor resection and colostomy. He was started on treatment with pembrolizumab (Keytruda®) in a dose of 400 mg intravenous every six weeks after tumor resection, and his symptoms appeared after five weeks of receiving the first immunotherapy infusion. His medical history was also significant for psoriasis and rheumatoid arthritis but unremarkable for thyroid disorders. The patient had no history of receiving thyroid medications or prior exposure to intravenous contrast media, over-the-counter iodine preparations, or biotin.

In the emergency department, the patient had a body temperature of 101.4°F (38.6°C), a heart rate of 110 beats per minute, and a blood pressure of 130/70 mmHg. He scored 45 points on the Burch-Wartofsky Point Scale. On examination, tenderness was elicited upon palpation of the anterior part of the neck. The patient was treated with intravenous fluids and broad-spectrum antibiotics. A workup for sepsis was performed and revealed negative blood culture results. His laboratory investigations demonstrated a low level of thyroid-stimulating hormone (TSH) (0.01 mIU/mL), which was lower than the TSH concentration reported at the oncology clinic before pembrolizumab (Keytruda®) administration (0.9 mIU/mL) (Table 1).

**Table 1:** Laboratory results BUN, blood urea nitrogen; A1C, glycated hemoglobin; TSH, thyroid-stimulating hormone

Laboratory parameters	Values	Normal range	Unit
White blood cell	8	4.5-11	10^9^/L
Hemoglobin	14	12-16	g/dL
Platelets	170	130-400	10^9^/L
Sodium	138	137-145	mmol/L
Potassium	4	3.5-5.2	mmol/L
Carbone dioxide	22	22-30	mmol/L
BUN	15	7-17	mg/dL
Creatinine	1	0.52-1.04	mg/dL
A1C	4.8	<5.7	%
TSH	0.01	0.5-5	mIU/L
Free T4	3.2	0.9-2.3	ng/dL
Anti-thyroglobulin antibody titer	130	<116	IU/mL
Anti-thyroid peroxidase antibody titer	25	<9	IU/mLco
Thyroid-stimulating immunoglobulin	0.9	<0.55	IU/L
Blood culture	No bacterial growth	No bacterial growth	

**Figure 1:**
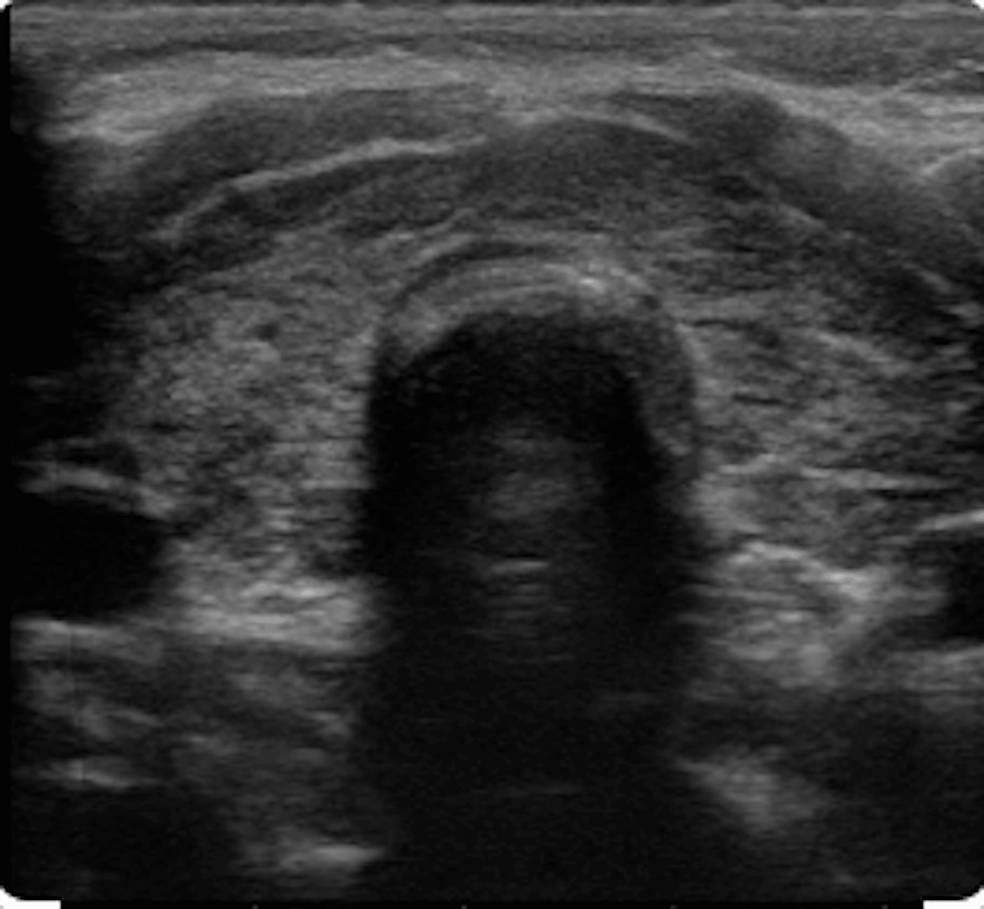
Thyroid ultrasound The image shows a heterogeneous thyroid gland, with a size of 40 mL

**Figure 2:**
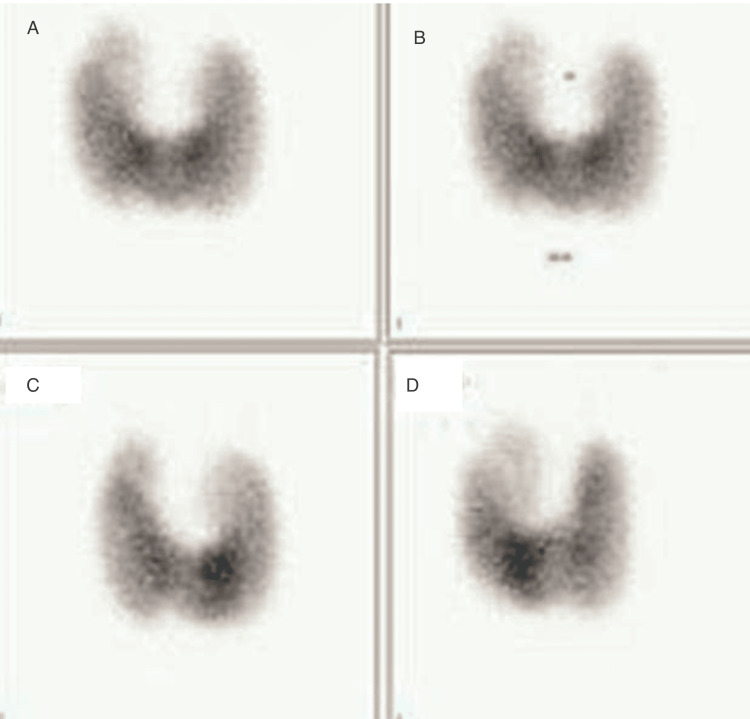
A radioactive iodine uptake test A, B, C, and D demonstrate increased contrast uptake by the thyroid gland (53% for 24-hour uptake)

A thyroid ultrasound was obtained, which revealed a heterogeneous thyroid gland consistent with thyroiditis (Figure 1). Antibody testing showed positive anti-thyroglobulin (Tg) antibodies, elevated anti-thyroid peroxidase (TPO) antibody titers, and thyroid-stimulating immunoglobulin (TSI). A radioactive iodine uptake test was ordered, demonstrating increased contrast uptake by the thyroid gland (Figure 2). These findings suggested that the cause of thyroid dysfunction in this patient was Graves’ disease. Based on the patient’s recent history of initiating pembrolizumab (Keytruda®) therapy and the absence of exposure to any other precipitating factors, his thyroid dysfunction was attributed to the immunotherapeutic agent, and a diagnosis of pembrolizumab-induced Graves’ disease was established. The patient’s symptoms improved after initiating intravenous fluid, intravenous Solu-Medrol 40 mg every eight hours for five days, metoprolol 25 mg tablet daily, and methimazole 10 mg every eight hours daily. And the patient was given an endocrinology referral upon discharge. At his one-month endocrinology visit, the patient reported that his symptoms had resolved.

## Discussion

Pembrolizumab (Keytruda®) is a humanized monoclonal IgG4κ antibody that acts as an immune checkpoint inhibitor (ICPi). It binds to the programmed cell death protein 1 (PD-1) receptor on T lymphocytes and blocks its interaction with the programmed death-ligand 1 (PD-L1) presented on the surface of tumor cells. The interaction between PD-1 and PD-L1 inhibits the T-cell response against tumor cells. Thus, by preventing this interaction, pembrolizumab restores T-cell-mediated antitumor immunity [4,5]. At present, pembrolizumab has been approved by the Food and Drug Administration (FDA) for the treatment of various types of cancer, including melanoma, non-small cell lung cancer (NSCLC), head and neck squamous cell carcinoma (HNSCC), renal cell carcinoma (RCC), and urothelial carcinoma [5,6]. On June 29, 2020, it was also approved as the first-line therapy in patients with microsatellite instability-high (MSI-H) or mismatch repair-deficient (dMMR) metastatic colorectal cancer [7].

Despite the remarkable survival benefits of pembrolizumab in patients with different types of cancer, it may be associated with developing some serious adverse effects. The most commonly reported adverse effects include fatigue, musculoskeletal pain, cough, dyspnea, skin rash, pruritus, nausea, diarrhea, abdominal pain, constipation, and decreased appetite. However, the more worrisome adverse effects are the immune-related adverse events (irAEs), which may include immune-mediated colitis, pneumonitis, encephalitis, nephritis, hepatitis, myocarditis, and endocrinopathies, such as thyroid disorders, type I diabetes, and hypophysitis [5,8]. Studies have shown that pembrolizumab therapy is associated with developing irAEs in 57%-79.5% of cases [9]. Thyroid dysfunction is the most common endocrine irAE seen in association with pembrolizumab. Results from the KEYNOTE-006 phase III clinical trial revealed that hypothyroidism occurred in up to 10.1% of the patients receiving pembrolizumab therapy. In comparison, hyperthyroidism was seen in up to 6.5% of the patients who received this immunotherapeutic agent [10].

Thyroid dysfunction in patients receiving pembrolizumab therapy typically presents as silent inflammatory thyroiditis, which develops within weeks to months after the initiation of treatment. Thyroiditis is biphasic and consists of transient thyrotoxicosis followed by secondary hypothyroidism. Graves’ disease (hyperthyroidism and/or Graves’ ophthalmopathy) secondary to pembrolizumab therapy has been rarely reported in the literature [4,11]. Peiffert et al. conducted a retrospective review of 243 patients who received treatment with immune checkpoint inhibitors (ICPis). Five out of the 243 patients were found to have ICPi-induced Graves’ disease. Among these five patients, only two had received pembrolizumab monotherapy. A review of the literature performed by the authors revealed only three other case reports of Graves’ hyperthyroidism [11]. To our knowledge, two additional cases of pembrolizumab-related Graves’ disease have been reported in the literature [12,13].

Graves’ disease is typically characterized by the development of hyperthyroidism and diffuse goiter. Ophthalmopathy may be present in around 50% of the patients [14]. Graves’ disease results from a failure of self-tolerance to the thyroid-stimulating hormone (TSH) receptor. The PD-1 pathway plays an essential role in peripheral tolerance. PD-1 is expressed on both B-cells and T-cells and can inhibit T-cell proliferation and activation, as well as B-cell receptor signaling and antigen-stimulated activation of B-cells. In this way, the PD-1 pathway causes the downregulation of immune responses and helps prevent the development of autoimmune diseases. Hence, it is hypothesized that an anti-PD-1 antibody, such as pembrolizumab, may induce Graves’ disease by inhibiting this pathway and activating the autoimmune system. However, further research is needed to understand the underlying mechanism entirely [15].

The role of thyroid autoantibodies in the pathogenesis of anti-PD-1-induced thyroid dysfunction remains unclear. While some studies have reported anti-thyroglobulin (Tg) and anti-thyroid peroxidase (TPO) antibodies associated with thyroid dysfunction induced by ICPi therapy, other studies have not been able to demonstrate this association. For instance, in a study conducted by Osorio and colleagues, anti-thyroid antibodies were present in eight out of 10 patients who developed thyroid dysfunction during pembrolizumab therapy [16]. It is important to note that Tg and TPO antibodies were also present in our patient. In contrast, in a retrospective analysis of patients treated with pembrolizumab at the Mayo Clinic, TPO antibodies were found to be present in 50% of the patients; however, the antibody titers were not elevated in patients who developed thyroiditis. In another study by Delivanis et al., TPO antibodies were absent in most patients treated with pembrolizumab who developed thyroid abnormalities. Based on these results, the authors concluded that an antibody-independent mechanism is likely responsible for thyroid dysfunction secondary to pembrolizumab therapy and may involve T-cell, natural killer (NK) cell, and/or monocyte-mediated pathways [17].

The general management of patients who develop adverse events related to immunotherapy involves the cessation of the offending agent and the initiation of immunosuppressants, such as corticosteroids [18]. According to the American Society of Clinical Oncology (ASCO) guidelines, patients with mild hyperthyroidism can continue ICPi therapy with close monitoring of TSH and free T4 levels every 2-3 weeks. Patients with moderate thyrotoxicosis usually require symptomatic treatment, while patients with severe symptoms may need treatment with anti-thyroid drugs or corticosteroids, along with the discontinuation of the ICPi [19].

Graves’ disease induced by pembrolizumab therapy is a severe yet rare immune-related adverse event (irAE) associated with this immunotherapeutic agent. We herein report the case of a patient who presented with fever, chills, nausea, vomiting, abdominal pain, and weight loss after receiving the first infusion of pembrolizumab. The suppressed TSH levels, results of thyroid ultrasonography, and increased radioactive iodine uptake helped establish the diagnosis of Graves’ disease. The time between the initiation of pembrolizumab therapy and the subacute onset of symptoms led us to identify pembrolizumab as the causative agent. This case report highlights the significance of a thorough evaluation to determine the cause of hyperthyroidism in patients receiving pembrolizumab, as an early diagnosis can result in the timely initiation of etiology-targeted management.

## Conclusions

Pembrolizumab (Keytruda®) is an immune checkpoint inhibitor (ICPi) that binds the PD-1 receptor on T-cells and blocks the PD-1/PD-L1 pathway. This results in the stimulation of T-cell-mediated antitumor immunity and can be beneficial in treating different types of malignancies. However, it may also result in the development of several immune-related adverse events (irAEs). Graves’ disease is a rarely reported irAE associated with this medication. Despite its rarity, physicians should consider pembrolizumab-induced Graves’ disease as a potential cause of thyrotoxicosis in patients receiving this medication. A high suspicion index must be maintained for this condition’s prompt diagnosis and rapid institution of treatment.
